# Novel Mutation in Chromosome 11p15.4 Causing Niemann-Pick Disease Type A in a Saudi Child

**DOI:** 10.7759/cureus.55883

**Published:** 2024-03-10

**Authors:** Adel M Al Shahrani, Walaa Asiri, Saad Ali M Alqarni, Lujaine M Al Murayeh

**Affiliations:** 1 Department of Pediatric Gastroenterology, Abha Maternity and Children Hospital, Abha, SAU; 2 Department of Pediatrics, Abha Maternity and Children Hospital, Abha, SAU; 3 College of Medicine, University of Bisha, Bisha, SAU

**Keywords:** niemann-pick disease, genetic disease, hepatosplenomegaly, acid sphingomyelinase deficiency, lysosomal storage diseases

## Abstract

Niemann-Pick disease (NPD) encompasses a minimum of three lysosomal storage diseases, all of which are inherited in an autosomal recessive manner. Acid sphingomyelinase (ASM) deficiency is the cause of NPD types A and B. ASM is the enzyme that hydrolyzes the sphingolipid sphingomyelin. An 18-month-old patient with progressive painless abdominal distension with organomegaly and neurological deficits presented to our hospital. Brain imaging and laboratory findings did not show anything, but there was a millstone growth delay. The diagnosis of NPD type A was confirmed by a genetic examination, which revealed a twofold change on chromosome 11p15.4 in the region encoding the sphingomyelin phosphodiesterase-1 (SMPD1) gene. The patient was followed up with no specific treatment, and signs of respiratory infections were later reported.

## Introduction

A lysosomal storage disease (LSD) known as Niemann-Pick disease (NPD) is brought on by variations in the sphingomyelin phosphodiesterase-1 (SMPD1) gene, which create an autosomal recessive condition. The condition was initially clinically characterized by pediatrician Albert Niemann in 1914, and its histological description was established by pathologist Ludwig Pick in 1927. Subsequently, in 1961 and 1966, Allen Crocker and Roscoe Brady divided that into four categories: A, B, C, and D. These categories are associated with defects in lysosomal proteins that cause improper transport of lipids inside cells, leading to neurological impairment and death [1,2].

A research released in 2021 on the Orphanet platform for rare illnesses and orphan medicines estimates that over 300 million individuals worldwide are thought to have a rare pathology [3]. LSDs are a class of genetic diseases that include at least 50 different cases. These diseases are caused by a lack of a specific lysosomal protein or activity or occasionally by non-lysosomal activities related to protein maturation or lysosomal biogenesis. One in 7,700 live births is the estimated general frequency, with a high incidence in the Ashkenazi community. While the p.Ala359Asp mutation that causes NPD type B has been found to have a carrier frequency of 1:105 in Chile, yielding a disease incidence of 1:44,960, there is no evidence available on the prevalence of the disease in Ecuador, Mexico, etc. [2]. Due to the misinterpretation of these uncommon conditions, reported epidemiological data are likely to be understated, which has an immediate impact on the effects of LSDs on the population, including Gaucher, Fabry, and Niemann-Pick [4].

There are currently two distinct entities of NPD, type A and type B, with intermediate forms falling under the first entity. This entity is known as acid sphingomyelinase-deficient NPD (ASM-deficient NPD) and is caused by mutations in the SMPD1 gene. Type D is a part of NPD type C (NPC), which arises from mutations in the NPC1 or NPC2 gene and is currently characterized as a disruption in cellular cholesterol transportation. In the brain, gangliosides are stored lipids that can cause various signs and symptoms based on the specific mutation they possess [5]. 

A child's hepatosplenomegaly and newborn cholestatic jaundice are the systemic symptoms. Along with the age of onset, they also depend on the following periods: early infantile (delay in developmental motor milestones), late infantile and juvenile (gait problems, falls, clumsiness, cataplexy, school problems), and in adult (ataxia and psychiatric disturbances); most affected patients may present with dysphagia, dysarthria, cerebellar ataxia, vertical supranuclear gaze palsy, and progressive dementia [5].

The value of clinical case presentations helps to internalize knowledge on rare diseases such as NPD. Therefore, even in cases where hepatomegaly or splenomegaly are not pathognomonic indications, an experienced doctor might assume this type of illness and order testing to confirm or rule out common conditions when a juvenile patient arrives with these symptoms. As a result, afflicted babies and their parents will greatly benefit from early identification and management before the onset of significant and irreversible signs.

## Case presentation

We describe the following prenatal history of our patient with NPD: normal birth, normal weight for gestational age, normal head circumference, normal weight of 2000 g, Apgar scores of 8-9, and normal Capurro of 39 weeks. The physical check-up was normal. The mother had no pre-existing medical conditions, no abnormalities, and no prior exposure to any harmful substances before pregnancy. No sudden deaths or cases of degenerative, chronic, or genetic diseases have occurred in her family.

**Figure 1:**
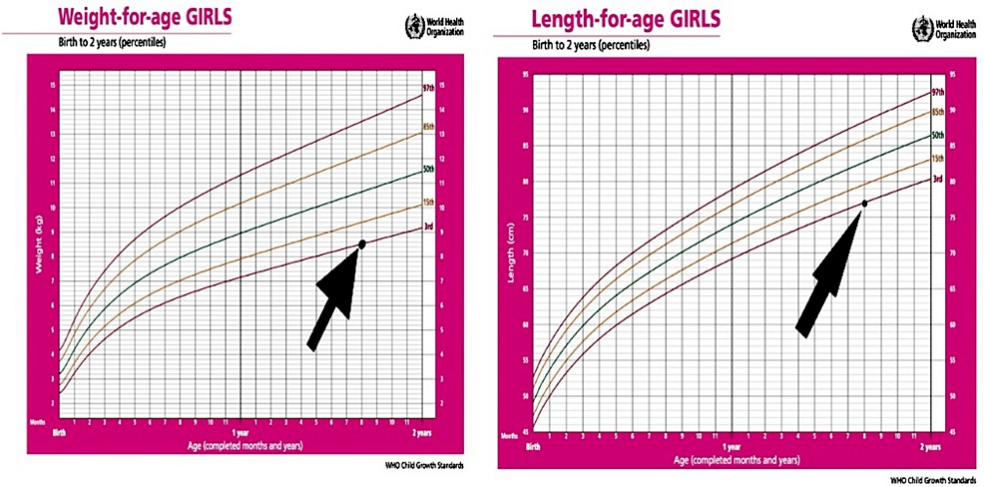
WHO child growth standard. WHO: World Health Organization

At 18 months of age, the patient presented progressive abdominal distension that did not relate to food or medications and was not associated with pain and progressive hypotonia, muscle weakness, milestone loss, spasticity, and rigidity. He was transferred from primary care to an outpatient clinic in the maternity and children's hospital. Anthropometric data at the time is shown in Figure 1.

**Figure 2:**
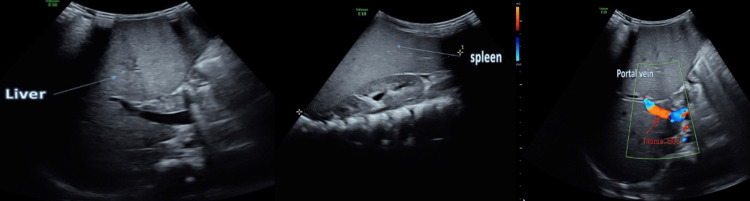
Doppler ultrasound reports hepatosplenomegaly.

The diagnosis of hepatosplenomegaly was confirmed by the results of the Doppler portal ultrasonography, which showed an enlarged liver measuring 13 cm and an enlarged spleen measuring 14.3 cm (Figure 2). A neurology team examined the patient and found nothing abnormal. In addition, there were no abnormalities detected by head computed tomography. 

Following a comprehensive examination of imaging and laboratory testing, infectious, hematologic, and neoplastic diseases were ruled out. Due to the high suspicion of metabolic storage illness, a medical meeting was held to decide whether to do an enzyme activity assay and molecular genetic research testing. It revealed a twofold mutation in the sequence that encodes the SMPD1 gene; it was found on chromosome 11p15.4, confirming the diagnosis of NPD type A (Table 1). 

**Table 1:** Molecular genetic studies show the abnormal sequencing analysis of SMPD1. OMIM: Online Mendelian Inheritance in Man; SMPD1: sphingomyelin phosphodiesterase-1

Gene and transcript	cDNA	Amino acid change	Zygosity	OMIM* phenotype (inheritance)
SMPD1 (NM_000543.5)	c.1616A>G	p.Tyr539Cys	Homozygous	Niemann-Pick disease, type B (AR) Niemann-Pick disease, type A (AR)

Laboratory tests were performed where complete blood count, electrolytes, ferritin coagulation times, and report values were shown in Table 2. Abnormal laboratory values were seen in the liver function test (Table 3). The serology of the viral markers revealed that hepatitis revealed negative hepatitis A virus, negative C-reactive protein, negative *Brucella abortus*, and negative *Brucella melitensis*. 

**Table 2:** Laboratory tests including complete blood count, electrolytes, and ferritin coagulation times report. Normal laboratory values, Department of Pediatrics, Abha Maternity and Children Hospital, Abha, Asir Region, Kingdom of Saudi Arabia* HGB: hemoglobin; WBC: white blood cell; RBC: red blood cell; K: potassium; Na: sodium; APTT: activated partial thromboplastin time; PT: prothrombin time; INR: international normalized ratio

Complete blood count	Lab values	Normal values*
HGB	10.4 g/dL	12-15
WBC count, 10^3^/uL	3.1	4-10
Neutrophil count, 10^3^/uL	1.2	2-7
Neutrophils %	38.9%	40-50
Lymphocytes %	57.4%	20-40
Eosinophils %	0.3%	1-6
Basophils %	0.5%	1-2
RBC count, 10^6^/uL	3.93	
Platelet count, 10^3^/uL	31	150-410
K	3.64 mmol/L	3.4-4.7
Calcium	8.4 mg/dL	8.8-10.8
Na	141.5 mmol/L	136-145
Ferritin	49.1 ng/mL	13-150
Coagulation time	Lab values	Normal values*
APTT	30.9 sec	26-40
PT	12.5 sec	11.5-15
INR	1.03%	0.9-1.1

**Table 3:** Liver function test reports Normal laboratory values, Department of Pediatrics, Abha Maternity and Children Hospital, Abha, Asir Region, Kingdom of Saudi Arabia* ALT: alanine aminotransferase; AST: aspartate aminotransferase

Liver function test	Lab values	Normal values*
ALT	192.6 U/L	0-50
AST	234.4 U/L	5-43
Total bilirubin	0.5 mg/dL	0.3-1.2
Direct bilirubin	0.1 mg/dL	0-0.50

During hospitalization, the patient got supportive therapy for gastrointestinal discomfort before being released with appropriate disease-prognosis advice. The child is presently two years and four months old, and his mother reports periodic slight fever and shortness of breath, which has been diagnosed as bronchiolitis. Our patient was subjected to a multidimensional and integrated management approach, characterized by the implementation of a broad array of interventions. This encompassed the administration of supportive treatment to address symptomatic concerns, alongside the initiation of physical and occupational therapy interventions, aimed at optimizing the patient's overall well-being and functional capacity.

## Discussion

The current case report showed an indirectly diagnosed case of NPD type A. A 1.5-year-old child with progressive unexplained painless abdominal distension showing signs of neurological deficits (progressive hypotonia, muscle weakness, loss of milestones, spasticity, and rigidity) was presented to a maternity and pediatric hospital. All of these reported neuroglial deficits in addition to the age of the child were consistent with NPD, mainly type A [6,7], which is diagnosed by exclusion as the hospital's neurological team reported no significant pathological findings. In addition, head computed tomography was clear of any abnormalities in addition to infectious, hematological, and tumor pathologies after reviewing the laboratory findings and images of the cases. Literature showed that patients may first present to general practitioners with nonspecific symptoms, the disease often remains undetected, or the diagnosis is made with a long delay [8]. Neurologic symptoms, which are typically characterized by hypotonia, seizures, and loss of deep tendon reflexes, are more common in cases of NPD type A and are not evident in other types [9]. While brain imaging is generally normal, which is the situation in our study case, distinctive findings include leukodystrophy, white matter signal hyperintensity in T2, and brain atrophy [10]. Additionally, the current cases showed hepatosplenomegaly that was diagnosed by the portal Doppler ultrasound. A genetic evaluation revealed that confirmation of NPD type A was provided by a twofold change in the sequence encoding the SMPD1 gene, which was found on chromosome 11p15.4. The association between 11p15.4 microdeletion and hemihypertrophy was presented in a case reported by Puvabanditsin et al. [11] that explains the associated neurological deficits among NPD cases. Later, the current case showed signs of respiratory infection. Respiratory involvement can be seen in all forms of NPD but has been seen most frequently in type B [12,13]. Furthermore, Niemann-Pick cells accumulate in the alveolar septa, bronchial walls, and pleura, possibly contributing to a gradually deteriorating restrictive pattern in the pulmonary function test [14,15]. All of these estimated symptoms were due to the accumulation of lipid-laden macrophages in various organs, such as the liver, spleen, bone marrow, organs of the central nervous system, and lung [14].

Our case also showed growth delay and rerated milestones, which was consistent with NPD types B and A/B [16]. There is evidence that the degree of organomegaly, decreased bone age, and decreased insulin growth factor 1 (IGF-1) are strongly correlated with the degree of growth delay. Additionally, liver dysfunction is part of the explanation for this delay [17]. Although bone and joint pain was absent in our case, the patient had a growth delay that was noticeable from the time of his initial appointment at 18 months of age; in addition to other classic symptoms of the disease, his low height and weight were consistent with NPD type A.

However, because of its genotype, there is a chance that when the illness worsens in the future, this change will become noticeable. The literature showed that patients may be predisposed to liver failure in childhood, which is the primary cause of death from this disease. Furthermore, this disease can cause death in early adulthood, primarily due to respiratory failure [18]. For the current study, no biopsy was performed, but a key indicator that points to NPD could be a bone marrow biopsy and a histopathological analysis of the organs that are frequently affected, such as the liver, spleen, and intestine [1].

To improve early diagnosis, we need to raise the awareness of NPD by physicians. This is because early detection of NPC is necessary for the administration of miglustat (Zavesca, Actelion Pharmaceuticals Ltd.), a medication that functions as a competitive inhibitor of the enzyme glucosylceramide synthase. Miglustat can extend survival by delaying the onset of neurological symptoms [19].

## Conclusions

Our case report highlights an indirectly diagnosed case of NPD type A in a 1.5-year-old child presenting with progressive abdominal distension and neurological deficits. The child exhibited symptoms consistent with NPD type A, including hypotonia, muscle weakness, loss of milestones, spasticity, rigidity, and hepatosplenomegaly. A genetic evaluation confirmed NPD type A through a twofold change in the sequence encoding the SMPD1 gene. Respiratory infection was also observed in this case, which is common in NPD and often seen in type B. The accumulation of lipid-laden macrophages in various organs, including the liver, spleen, bone marrow, central nervous system, and lung, contributed to the observed symptoms. Growth delay and delayed milestones were evident, possibly associated with organomegaly, decreased bone age, decreased IGF-1, and liver dysfunction. Ophthalmic signs, typically seen in NPD type A, were not present in this case but may become noticeable as the disease progresses due to the genotype.

Early diagnosis of NPD is crucial for timely intervention with miglustat, a medication that delays the onset of neurological symptoms by inhibiting the enzyme glucosylceramide synthase. To improve early detection, raising awareness among physicians is essential. Biopsy and histopathological analysis of affected organs, such as the liver, spleen, and intestine, can also aid in confirming the diagnosis. NPD type A poses a significant risk of liver failure in childhood and respiratory failure leading to death in early adulthood. This case underscores the importance of early diagnosis and intervention to improve patient outcomes and extend survival in individuals with NPD type A. Further research and awareness are needed to enhance the understanding and recognition of this rare disease among healthcare professionals.
